# Recognition and treatment of attention deficit-hyperactivity disorder in patients with treatment-resistant burning mouth syndrome: a retrospective case study

**DOI:** 10.3389/fpain.2025.1536584

**Published:** 2025-04-23

**Authors:** Kaori Takahashi, Satoshi Kasahara, Miwako Takahashi, Taito Morita, Naoko Sato, Toshimitsu Momose, Ko Matsudaira, Shin-Ichi Niwa, Kanji Uchida, Toshiyuki Handa, Tatsuya Ichinohe, Ken-ichi Fukuda

**Affiliations:** ^1^ Department of Dental Anesthesiology, Tokyo Dental College, Tokyo, Japan; ^2^ Department of Anesthesiology and Pain Relief Center, The University of Tokyo Hospital, Tokyo, Japan; ^3^ Department of Pain Medicine, Fukushima Medical University School of Medicine, Fukushima, Japan; ^4^ Department of Molecular Imaging and Theranostics, Institute for Quantum Medical Science, National Institutes for Quantum Science and Technology, Chiba, Japan; ^5^ Nursing Department, The University of Tokyo Hospital, Tokyo, Japan; ^6^ Institute of Engineering Innovation, School of Engineering, The University of Tokyo, Tokyo, Japan; ^7^ Department of Orthopaedic Surgery, Tailor Made Backpain Clinic, Tokyo, Japan; ^8^ Department of Psychiatry, Aizu Medical Center, Fukushima Medical University, Fukushima, Japan; ^9^ Division of Special Needs Dentistry and Orofacial Pain, Tokyo Dental College, Tokyo, Japan

**Keywords:** burning mouth syndrome, nociplastic pain, attention deficit hyperactivity disorder, methylphenidate, multidisciplinary approach, single-photon emission computed tomography, frontal hypoperfusion, precuneal hyperperfusion

## Abstract

**Introduction:**

Burning mouth syndrome (BMS) is an idiopathic oral pain disorder characterized by burning sensations and dysesthesia, often complicated by psychosocial factors and psychiatric comorbidities, necessitating a multidisciplinary approach. BMS, classified as nociplastic pain (NcplP), frequently involves central sensitization. Attention-deficit/hyperactivity disorder (ADHD), a neurodevelopmental disorder, is commonly comorbid with NcplP, and ADHD-targeted treatment has shown efficacy in NcplP management. However, the role of ADHD diagnosis and treatment on BMS and associated brain function abnormalities remains unexplored. Therefore, we aimed to investigate the prevalence of ADHD comorbidity and its assessment using ADHD scales and the therapeutic efficacy of an ADHD-focused algorithm, including pre- and post-treatment cerebral blood flow single-photon emission computed tomography (SPECT) results, in patients with treatment-resistant BMS referred from the outpatient clinic of dental psychosomatic specialists at a tertiary care institution for multidisciplinary treatment.

**Methods:**

We retrospectively analyzed data from 14 patients with treatment-resistant BMS who received multidisciplinary care, including psychiatric evaluation and SPECT imaging. Clinical assessments included the Conners' Adult ADHD Rating Scale (CAARS-S and CAARS-O), Pain Numerical Rating Scale, Hospital Anxiety and Depression Scale, and Pain Catastrophizing Scale. Algorithm-based pharmacotherapy using ADHD-effective medications (methylphenidate, atomoxetine, guanfacine, aripiprazole, venlafaxine, and duloxetine) was administered.

**Results:**

ADHD was diagnosed in 13 patients (92.9%), with 57.2% exhibiting borderline or clinical-level symptoms. Clinically significant improvements were observed in all clinical scales among the 10 patients who completed algorithm-based treatment. Brain perfusion SPECT identified hypoperfusion in the frontal lobe and hyperperfusion in the perigenual anterior cingulate cortex, insular cortex, posterior cingulate gyrus, and precuneus in 90% of cases, with improvements noted following treatment.

**Conclusions:**

ADHD is frequently comorbid in patients with treatment-resistant BMS, and ADHD-targeted pharmacotherapy may help alleviate pain, cognitive dysfunction, and brain perfusion abnormalities. These findings suggest that ADHD screening, diagnosis, and multidisciplinary management involving psychiatrists could play a crucial role in optimizing clinical outcomes in patients with BMS.

## 1 Introduction

Burning mouth syndrome (BMS) is defined as oral pain of unknown origin, characterized by a burning or abnormal sensation lasting at least 2 h daily for a minimum of 3 months (1). The global prevalence of BMS is estimated to range from 0.1%–3.9%, with postmenopausal women aged 50–70 years being the most affected demographic (2, 3). The burning pain and numbness in the oral cavity—including the tongue, lips, palate, and gums (4)—typically worsen throughout the day, peaking in the evening (5). Despite its clinical impact, BMS remains challenging to diagnose and treat, with a spontaneous remission rate as low as 3%–4% within 5–6 years of diagnosis (6). Its pathogenesis is not fully understood; however, its etiology in many patients has been suspected to involve local, systemic, and/or psychological contributions (4). Dysfunction of dopaminergic pathways in the central nervous system (CNS) has been implicated as one of the systemic factors involved (7). BMS is frequently associated with depression, and reduced cerebral blood flow (CBF) in the left parietal and temporal lobes has been observed in patients with both BMS and depression (8). Indeed, BMS is now recognized as closely linked to psychosocial factors and psychiatric comorbidities. Addressing the psychogenic component of the pain requires multidisciplinary research and treatment approaches incorporating the expertise of psychologists and psychiatrists (4, 9).

In 2017, pain traditionally categorized as psychogenic or related to somatoform disorders was redefined as nociplastic pain (NcplP) (10), which is now recognized as a third pain type alongside nociceptive and neuropathic pain. NcplP is thought to involve plastic changes in nociceptive central neural circuits, leading to central sensitization and amplification of external stimuli (11). A key clinical feature of NcplP is its frequent association with CNS symptoms, including hyperalgesia, fatigue, sensory hypersensitivity to sound or light, sleep disturbances, mood disorders, and cognitive dysfunctions, such as impaired attention and memory. Additionally, it is often influenced by various psychosocial factors (12).

Recent studies have reported that NcplP disorders—such as fibromyalgia (13–15), chronic low back pain (16–20), idiopathic orofacial pain (19, 21–23), temporomandibular joint disorders (24, 25), chronic chest pain (26), chronic abdominal pain (27–29), and irritable bowel syndrome (30)—frequently coexist with attention deficit-hyperactivity disorder (ADHD). ADHD has been suggested to contribute to the development of central sensitization and cognitive dysfunctions, including attention deficits and sensory overactivity, observed in NcplP disorders (31). Notably, when ADHD coexists with NcplP, symptoms of pain and related CNS dysfunctions, including cognitive impairments, have been shown to improve with ADHD medications (18, 19, 21–23, 28, 32–34). Furthermore, these medications have demonstrated the ability to modulate cerebral blood flow, improving reduced perfusion in the prefrontal cortex, increasing perfusion in the precuneus, and addressing blood flow imbalances in the anterior cingulate and insular cortices—regions collectively known as the pain matrix (18, 19, 28, 32). These findings suggest that ADHD medications suppress excessive activity in the default mode network (DMN) and enhance activity in the central executive network (35), making them a promising new treatment option for NcplP, which is often refractory to conventional therapies (36).

A previous study reported that 72.5% of patients with refractory NcplP had comorbid ADHD (33). BMS is now considered a representative NcplP disorder; however, no published reports exist on the prevalence of ADHD comorbidity in BMS or the potential efficacy of ADHD medications in improving BMS symptoms and related CNS dysfunctions. Additionally, no studies have investigated changes in CBF in patients with BMS before and after ADHD medication intervention.

In this study, we aimed to investigate the prevalence of ADHD comorbidity and its assessment using ADHD scales and the therapeutic efficacy of an ADHD-focused algorithm, including pre- and post-treatment CBF single-photon emission computed tomography (CBF-SPECT) results, in patients with treatment-resistant BMS referred from the outpatient clinic of dental psychosomatic specialists at a tertiary care institution for multidisciplinary treatment.

## 2 Materials and methods

### 2.1 Study design, setting, and patients

In this retrospective study, we enrolled 103 patients with BMS aged between 18 and 90 years who visited Tokyo Dental College Hospital between May 2020 to August 2022. Of these, 20 showed intractable symptoms despite treatment with clonazepam, capsaicin, pregabalin, amitriptyline, gargle liquid, and/or low-level laser therapy (LLLT) after ≥6 months. Fourteen of these 20 patients were referred to the Department of Anesthesiology and Pain Center, University of Tokyo Hospital. We retrospectively analyzed the records of these 14 patients who presented with tongue pain between July 2020 and August 2022.

### 2.2 Inclusion and exclusion criteria

Eligible patients were aged ≥ 18 years, diagnosed with BMS, and exhibited no improvement after ≥ 6 months of treatment (*n* = 14). Exclusion criteria encompassed secondary BMS or symptom remission following treatment (*n* = 83), and the wish not to be referred to the Pain Centre (*n* = 6). Severe psychiatric conditions (depression, suicidal ideation, or psychosis) impairing reality judgment or inducing manic states were not included in this study.

### 2.3 Diagnostic assessments

#### 2.3.1 BMS diagnosis and treatment

In this study, we defined an intraoral burning or dysesthetic sensation, recurring daily for >2 h for >3 months, without evident causative lesions on clinical examination and investigation, as described in the International Classification of Orofacial Pain—first edition (ICOP-1), as the diagnostic criterion for BMS. Currently, no definitive treatment for BMS is available; however, it is treated using methods consistent with those for neuropathic pain and NcplP. Reports have shown the effectiveness of clonazepam, capsaicin, pregabalin, and amitriptyline as pharmacological therapy for BMS (37).

#### 2.3.2 ADHD diagnosis

Adult ADHD was assessed using the Conners’ Adult ADHD Rating Scale Self-Report (CAARS-S) and Observer-Report (CAARS-O) (38). These validated instruments comprise 66 items across eight subscales, leveraging T-scores based on a standardized, age-matched population. T-scores > 65 were considered indicative of clinically significant symptoms, while scores between 60 and 65 were categorized as borderline. ADHD traits such as “frequent careless mistakes”, “difficulty with organization”, or “impatience” were evaluated across average, borderline, and clinical levels.

A psychiatrist (S.K.) confirmed ADHD diagnoses based on the Diagnostic and Statistical Manual of Mental Disorders, Fifth Edition (DSM-5) criteria (39), and the Diagnostic Interview for ADHD in Adults 2.0 (DIVA 2.0), a semi-structured interview (40). The DIVA 2.0 exemplifies dysfunction in daily activities due to ADHD symptoms from childhood through adulthood across 18 diagnostic criteria within five domains: work/education, romantic/family relationships, social interactions, leisure/hobbies, and self-confidence/self-image. DSM-5 criteria require ≥ 5 of 9 inattention or hyperactivity/impulsivity symptoms for individuals aged ≥ 17 years. ADHD subtypes were classified as predominantly inattentive, predominantly hyperactive-impulsive, or combined. Other comorbid psychiatric disorders were also differentiated based on the DSM-5 diagnostic criteria.

#### 2.3.3 Assessment of psychosocial factors

The Multidimensional Pain Inventory (MPI) (41, 42) was used to assess psychosocial factors, as it contributes to predicting treatment responsiveness in patients with chronic pain. The MPI calculates a Dysfunctional (DYS) score, which reflects the tendency of family members to exhibit overprotective behaviors that reinforce the patient's pain, and an Interpersonally Distressed (ID) score, which indicates the patient's perception of being blamed by their family. Based on the balance of these scores, patients are classified into one of the following three categories: DYS, ID, or Adaptive Coper (AC). If a response does not fit into any of these three categories, it is classified as Anomalous.

In the DYS category, a symbiotic relationship tends to develop between the patient and their family, making family conflicts less apparent. In contrast, patients in the ID category tend to feel blamed by their families and have a heightened awareness of family relationship conflicts. Patients classified as AC experience conflicts with their families but can maintain an appropriate interpersonal distance. Patients in the DYS category are considered to respond well to family-involved operant behavioral therapy, those in the ID category to assertiveness training, and those in the AC category to education on pain self-management for symptom improvement (43).

#### 2.3.4 Pain assessment

Pain duration was measured as the time (in months) from BMS onset to the initial clinic visit. Pain intensity was evaluated using the Numerical Rating Scale (NRS) (44), with a minimum clinically important difference (MCID) of ≥2 points (45).

#### 2.3.5 Mood state assessment

Mood disturbances, including anxiety and depression, were assessed using the Hospital Anxiety and Depression Scale (HADS) (46). Each subscale ranged from 0 to 21, with scores ≥ 11 indicating clinical levels (47). The MCID was set at 1.5 points (48).

#### 2.3.6 Pain catastrophizing

Pain-related catastrophic thinking was assessed using the Pain Catastrophizing Scale (PCS) (49), which measures the degree to which individuals amplify or ruminate on pain experiences. Scores range from 0 to 52, with scores ≥ 30 indicating chronic pain within the 75th percentile. The MCID for PCS was set at 6.48 points (50).

#### 2.3.7 BMS severity

BMS severity was evaluated using the Clinical Global Impression Severity (CGI-S) scale (51), assessing the condition's impact on daily activities and cognitive functions (anxiety, depression, insomnia, attention deficits, and sensory sensitivity). Scores range from 1 to 7, where 1 = normal, 2 = borderline illness, 3 = mildly ill, 4 = moderately ill, 5 = markedly ill, 6 = severely ill, and 7 = extremely ill.

### 2.4 Medication algorithm

**Figure 1:**
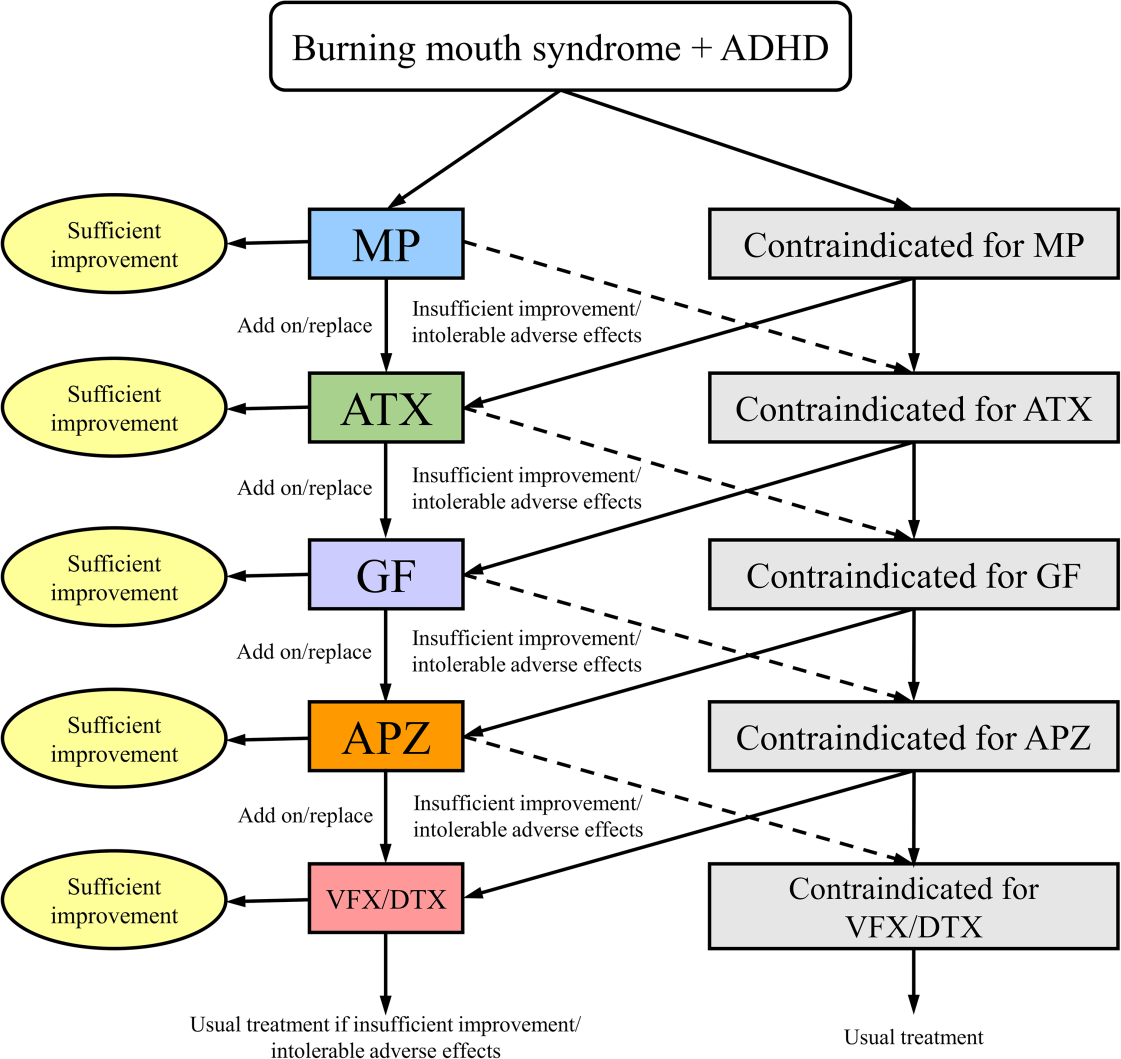
Algorithm for pharmacological management of patients with BMS and comorbid ADHD. ADHD, attention-deficit/hyperactivity disorder; APZ, aripiprazole; ATX, atomoxetine; DXT, duloxetine hydrochloride; GF, guanfacine; MP, methylphenidate; VFX, venlafaxine hydrochloride.

The pharmacotherapy algorithm for ADHD (52, 53) is shown in Figure 1. For patients without contraindications to pharmacological treatment, the first-line medication administered was the ADHD stimulant methylphenidate (MP). If MP does not achieve sufficient improvement or causes intolerable side effects, patients either transition to combination therapy with MP and the selective norepinephrine reuptake inhibitor atomoxetine (ATX) or switch to ATX monotherapy. Should ATX fail to provide adequate improvement or result in intolerable side effects, patients either receive combination therapy with the α2 agonist guanfacine (GF) or switch to GF monotherapy. If GF administration does not yield satisfactory outcomes, patients either receive combination therapy with aripiprazole (APZ) or switch to APZ monotherapy. APZ, a partial agonist of dopamine D2 receptors and dopamine system stabilizer, can modulate dopamine activity, either by enhancing or suppressing it as needed. Suppose APZ fails to provide sufficient improvement or causes intolerable side effects. In that case, treatment progresses to combination therapy with the serotonin-norepinephrine reuptake inhibitors venlafaxine (VFX) or duloxetine (DXT) or switches to VFX/DXT monotherapy. In cases where ADHD diagnostic criteria were not met, the algorithm initiated treatment with APZ. Treatment effects on NRS, HADS, and PCS scores, as well as CBF measurements, were evaluated 2 months after medication adjustments, provided there was adequate improvement without adverse effects.

This pharmacotherapy algorithm was developed based on the following considerations. First, MP was selected as the first-line medication because it is recommended as a first-line treatment for ADHD in various national ADHD treatment guidelines (54). Furthermore, given that the participants in this study had been suffering from BMS for an average of 6.1 years without improvement, a rapid-acting medication was considered beneficial for enhancing patient motivation and adherence to treatment. Other ADHD medications that are not rapid-acting include ATX and GF. However, in Japan, a generic version of ATX is available, whereas GF does not have a generic version and is relatively expensive. To reduce the financial burden on patients, ATX was designated as the second-line treatment, whereas GF was administered as the third-line option. For the fourth-line treatment, APZ was selected based on its demonstrated efficacy against ADHD (53), chronic pain (55), BMS (56), and idiopathic oral facial pain (21). For the fifth-line treatment, VFX/DXT was selected based on the evidence that, although classified as antidepressants, both VFX and DXT have been shown to be effective in treating ADHD (53) and chronic pain (28, 57).

### 2.5 CBF-SPECT imaging

CBF was assessed using SPECT imaging with commercially available ^99m^Tc-labeled ethyl cysteinate dimer (ECD; PDRadiopharma Inc., Chuo-ku, Japan). Patients were intravenously administered a 740-MBq (20 mCi) dose of ECD while resting supine with their eyes closed in a quiet environment. Approximately 5 min post-injection, a 30-min scan was conducted using a triple-head SPECT system (GCA-9300R; Cannon Medical Systems, Otawara, Japan) equipped with a high-resolution fan-beam collimator, achieving a spatial resolution of 7.2 mm full-width at half-maximum (FWHM).

Images were reconstructed using filtered back-projection with Butterworth and Ramp filters and collected in a 128 × 128 × 89 matrix with a voxel size of 1.72 × 1.72 × 3.44 mm. To minimize high-frequency noise, a 6-mm FWHM Gaussian filter was applied. The resulting images were rendered in axial, coronal, and sagittal views with rainbow color mapping. Two nuclear medicine experts (M.T., with approximately 20 years of experience, and T.M., with approximately 40 years of experience) independently conducted a qualitative visual analysis of the images to identify characteristic findings.

### 2.6 Statistical analysis

Changes in NRS, HADS, and PCS scores before and after treatment were evaluated using the Wilcoxon signed-rank test. Statistical significance was set at *P* < 0.05, with Bonferroni correction applied for multiple comparisons. Data analyses were performed using SPSS Statistics v25 (IBM Corp., Armonk, NY, USA).

### 2.7 Ethics approval and consent to participate

This study was approved by the University of Tokyo Hospital Research Ethics Committee (approval number 3,678). Verbal informed consent was obtained during the initial visit, followed by written informed consent for publication. Participants were informed of their right to decline participation or withdraw from the study at any time.

## 3 Results

### 3.1 Clinical characteristics

The demographic and clinical characteristics of the 14 participants (2 males, 12 females; mean age: 60.0 ± 16.3 years) are summarized in Supplementary Table S1. The average pain duration was 73.1 ± 71.5 months (approximately 6.1 years). The mean CGI-S score was 3.9 ± 1.1, indicating a range from mildly ill to markedly ill.

Based on the MPI classification of psychosocial factors, four patients (28.6%) were categorized as DYS, five (35.7%) as ID, four patients (28.6%) as AC, and one (7.1%) as Anomalous.

Previous pharmacological treatments for pain management included tricyclic antidepressants (64.3%), pregabalin (28.6%), clonazepam (14.3%), and other agents (7.1% each), such as anticonvulsants, tramadol hydrochloride, and acetaminophen. Furthermore, sleep medications (21.4%) and VFX were used as treatments for psychiatric conditions. Psychiatric treatment histories were noted in 5 participants (35.7%), including depression (21.4%), adjustment disorder (7.1%), and insomnia (7.1%). None of these treatments significantly alleviated BMS symptoms.

### 3.2 Assessment and diagnosis of ADHD

Thirteen participants (92.9%) met DSM-5 criteria for ADHD. ADHD subtypes were: predominantly inattentive (23.1%, 3 patients), predominantly hyperactive-impulsive (30.7%, 4 patients), and combined type (46.2%, 6 patients). Comorbid psychiatric conditions were present in five patients (35.7%), including autism spectrum disorder (28.6%, 4 patients) and depression (7.1%, 1 patient). ADHD symptoms assessed using the CAARS revealed that 28.6% of patients displayed clinical-level symptoms, 28.6% exhibited borderline symptoms, and 57.2% had ADHD symptoms at or above the borderline level. The highest CAARS subscale scores for each patient are detailed in Supplementary Table S1.

### 3.3 Medication regimens and outcomes

**Figure 2:**
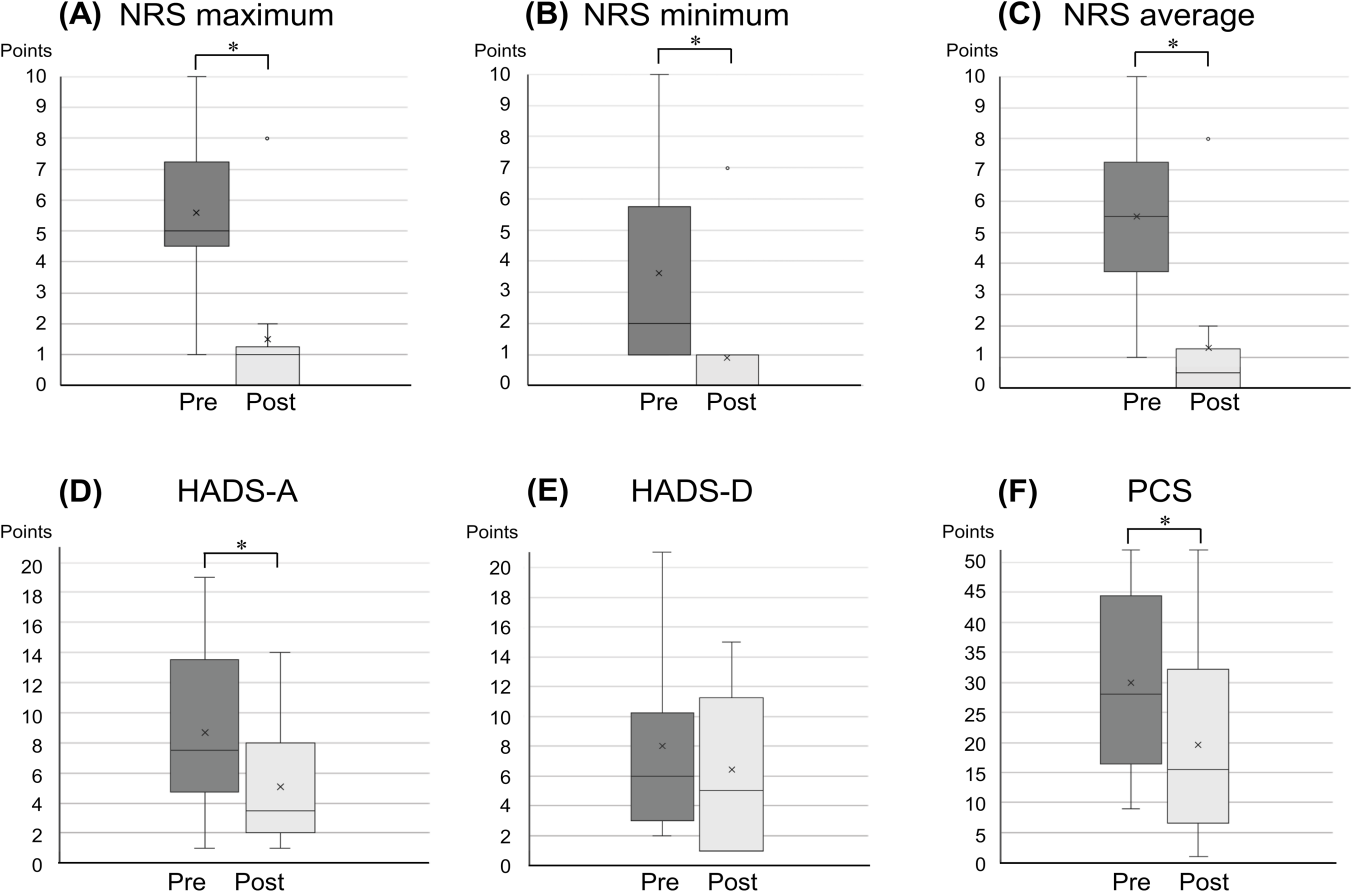
Changes in pain intensity and related symptoms pre- and post-treatment. Panels **(A–F)** illustrate the changes in pain NRS maximum, pain NRS minimum, pain NRS average, HADS-A, HADS-D, and PCS scores, respectively, pre- and post-treatment. *P < 0.05. HADS-A/D, hospital anxiety and depression scale anxiety and depression; NRS, numerical rating scale; PCS, Pain Catastrophizing Scale.

Four participants declined pharmacotherapy or discontinued early, opting for outpatient cognitive-behavioral therapy with S.K. The remaining 10 participants followed the study's pharmacotherapy algorithm. Post-treatment changes in pain-related and psychological scale scores are presented in Figure 2 and Table 1. No serious adverse events were reported. The observed improvements included: maximum pain NRS score decreased by 4.1 ± 1.0 points, minimum pain score by 2.7 ± 0.9 points, and mean pain score by 4.2 ± 1.0 points. Anxiety (HADS-A) scores improved by 3.6 ± 1.2 points, depression (HADS-D) scores by 1.6 ± 1.1 points (non-significant), and PCS scores by 10.3 ± 2.5 points. All mean changes exceeded the MCID.

**Table 1:** Changes in pain and pain-related symptoms before and after treatment.

Measure	N	Pre	Post	Mean difference	95% CI	*P*	Corrected P
NRS maximum	10	5 (4.5, 7.25)	1 (0, 1.25)	4.1	1.8–6.4	0.0078	<0.05
NRS minimum	10	2 (1, 5.75)	0 (0, 1)	2.7	0.6–4.8	0.0039	<0.05
NRS average	10	5.5 (3.75, 7.25)	0.5 (0, 1.25)	4.2	2.0–6.4	0.0078	<0.05
HADS-A	10	7.5 (4.75, 13.5)	3.5 (2, 8)	3.6	1.0–6.2	0.0078	<0.05
HADS-D	10	6 (3, 10.25)	5 (1, 11.25)	1.6	−0.9–4.1	0.2188	1.00
PCS	10	28 (16.5, 44.5)	15.5 (6.5, 32.25)	10.3	4.5–16.1	0.0078	<0.05

Median (25th and 75th percentile) values of pre- and post-treatment variables are shown. CI, confidence interval; HADS-A/D, hospital anxiety and depression scale—anxiety and depression; NRS, numerical rating scale; PCS, pain catastrophizing scale. *P* values were corrected by multiplying by the number of tests for multiple testing within the table (i.e., Bonferroni correction).

Among the 10 patients, the medications used included APZ (6 patients; 60.0%), MP (5 patients; 50.0%), ATX (3 patients; 30.0%), VFX (3 patients; 30.0%), GF (1 patient; 10.0%), and DXT (1 patient; 10.0%). Monotherapy was employed in 60.0% of cases, while 40.0% received combination therapy. Mean monotherapy doses were: MP, 18 mg/day; ATX, 20 mg/day; APZ, 5.8 ± 4.6 mg/day; VFX, 225 mg/day; and DXT, 20 mg/day.

### 3.4 CBF-SPECT results

**Figure 3:**
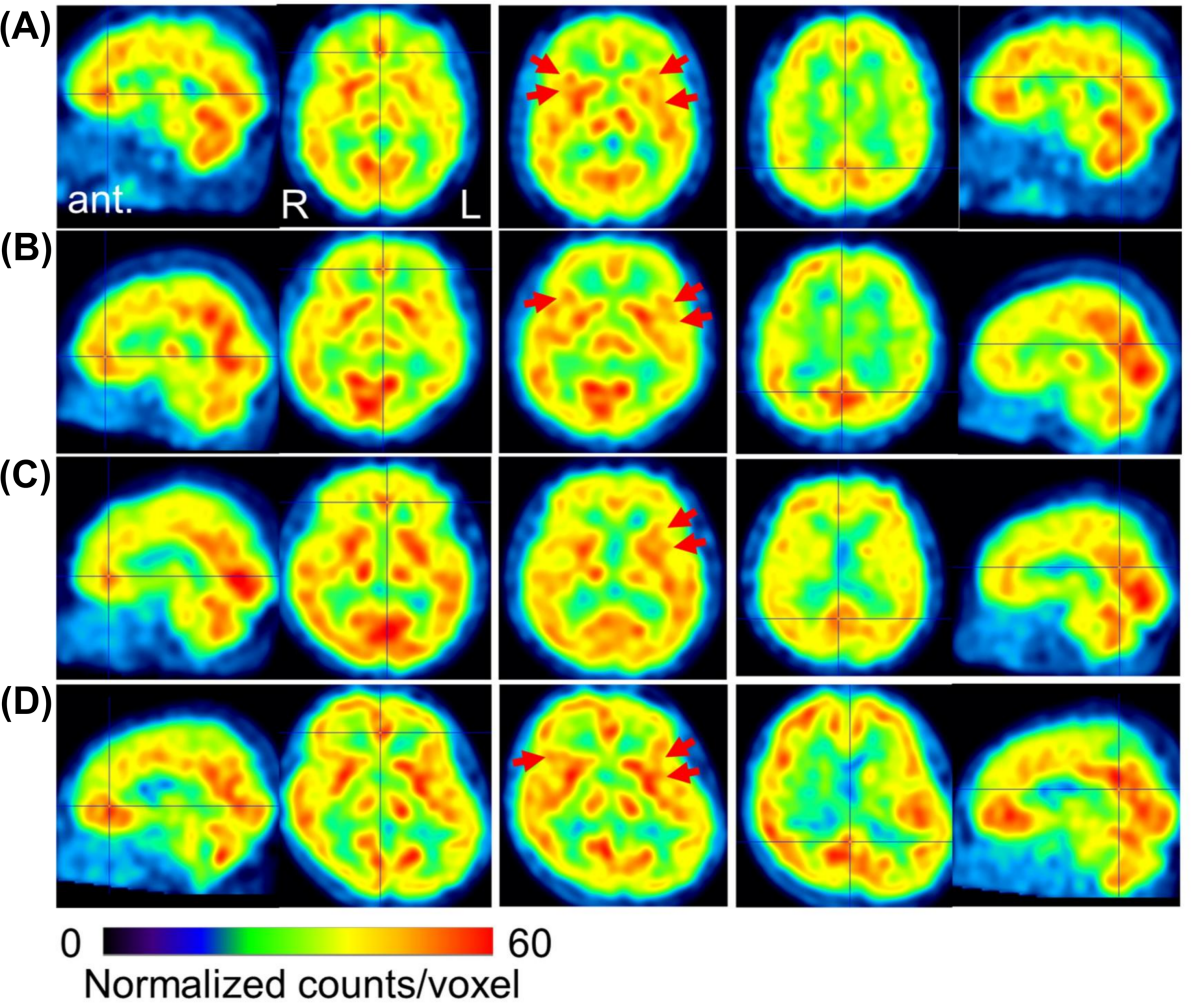
Representative cerebral blood flow SPECT images before treatment. Voxel values are normalized to average counts-per-voxel, with a cerebellar reference count of 50. The color bar represents count values ranging from 0 to 60. Panels **(A–C)** show images from patients #4, #5, and #12, respectively (all with a CGI-S score of 5), exhibiting focal hyperperfusion in the perigenual anterior cingulate cortex (crossbar) and the insular cortex (red arrows), alongside hypoperfusion in other frontal regions. Panel **(D)** displays images from patient #6 (CGI-S score of 2), who exhibited preserved CBF in the frontal and parietal lobes. The sagittal and axial views on the left indicate focal hyperperfusion, while the central axial views highlight increased perfusion in the insular cortex. The sagittal and axial views on the right show perfusion in the precuneus and posterior cingulate cortex. SPECT, single-photon emission computed tomography; CGI-S, clinical global impression severity; ant, anterior; R right; L, left.

Abnormal CBF-SPECT findings included hyperperfusion in the bilateral perigenual anterior cingulate cortex (pgACC), insular cortex, precuneus, and posterior cingulate cortex (PCC), with relative hypoperfusion in other frontal regions. The representative case's brain perfusion images are shown in Figure 3. These abnormalities were observed in all patients except Patients 3 and 6. Patient 6 exhibited preserved frontal regions, minimizing abnormal findings, while Patient 3 showed predominant frontal lobe hypoperfusion without pgACC or insular perfusion. Details for each patient are listed in Supplementary Table S1.

Pre- and post-treatment CBF-SPECT evaluations were available for six patients (Patients 1, 4, 6, 11, 13, and 14) with a mean interval of 18 ± 7 months (range: 7–25 months). While no significant differences were observed between pre- and post-treatment images, abnormalities appeared less severe following ADHD medication therapy.

## 4 Discussion

This study highlights three key findings in the field of psychiatry. First, a high comorbidity rate of ADHD (92.9%, 13/14 patients) was observed among individuals with refractory BMS. Second, a pharmacotherapy algorithm effectively alleviated pain, anxiety, depression, and pain-related catastrophic thinking in these patients. Third, pre-treatment SPECT imaging in 10 patients revealed reduced frontal lobe CBF in 90.0% of cases, with localized hyperperfusion commonly identified in the pgACC, insula, precuneus, and PCC. Among the six patients who underwent post-treatment SPECT, 83% demonstrated improved CBF abnormalities, achieving a distribution closer to that of healthy individuals.

To our knowledge, this study is the first to report a high diagnostic rate of ADHD in patients with refractory BMS. The findings suggest that ADHD may frequently coexist in patients with BMS. The etiopathogenesis of primary BMS remains unclear and is probably of multifactorial origin (4). In this study, we targeted patients with treatment-resistant BMS who did not show improvement with pharmacotherapy, mouthwashes for pain relief, or LLLT, a homeopathic approach. Anxiety and depressive states are prominent complaints in adult ADHD, with over 80% of cases estimated to be overlooked, even in psychiatric clinical practice (58). Since BMS treatment is often managed by dentists or pain clinicians unfamiliar with ADHD management, it can be inferred that comorbid ADHD is frequently unrecognized. This study's results offer a new perspective on BMS in clinical and research settings. The tendency of patients with BMS to seek invasive procedures or narcotics for immediate pain relief or to engage in doctor shopping may reflect the impulsivity associated with ADHD and the challenges in awaiting gradual results (21).

The reported comorbidity rates of chronic pain and ADHD include approximately 83.3% in treatment-resistant idiopathic orofacial pain (21), 72.5% in treatment-resistant chronic pain (33), 80.2% among psychiatric outpatients with chronic pain (59), and 80% in fibromyalgia (60). The ADHD comorbidity rate in this study aligns with these figures but is comparatively higher. However, the prevalence of ADHD in fibromyalgia has varied, with Reyero et al. reporting 32.3% (61) and Yilmaz and Tamam reporting 29.5% (62). These variations highlight the influence of the study population and methodology.

One factor contributing to the high comorbidity rate of ADHD in this study was the systematic and comprehensive evaluation of ADHD symptoms using the CAARS-S/O, which incorporated input from patients and their family members. Gathering information from multiple sources is crucial for accurate ADHD diagnosis, and combining self-reported data and observer-reported data is recommended (38). Additionally, using the CAARS-O when collecting information from family members allows for a thorough review of all 18 diagnostic criteria for ADHD as outlined in the DSM-5. Therefore, the use of the CAARS-O in combination with structured interviews for adult ADHD diagnosis is recommended. Furthermore, tailoring questions based on the responses to the CAARS-O helps focus inquiries during patient interviews, making it easier to extract relevant information, which is highly useful for diagnosis (63).

The ADHD comorbidity rate of 92.9% in this study may appear remarkably high; however, it likely reflects the systematic use of the CAARS-S/O, which allowed for precise identification of ADHD-related episodes. We recommend collaboration with psychiatrists and the use of the CAARS-S and CAARS-O for thorough ADHD screening in patients with chronic pain, including BMS.

Among the 14 patients with BMS screened using CAARS, four (28.6%) exhibited ADHD symptoms at a clinical level, and another four (28.6%) demonstrated borderline symptoms. ADHD traits at borderline levels, even below diagnostic thresholds, have been shown to affect pain (59). Thus, including borderline traits in ADHD evaluations of patients with chronic pain is essential. Our findings support the high diagnostic rate of ADHD in this population.

The ADHD subtype distribution in this study was 23.1% predominantly inattentive, 30.8% predominantly hyperactive-impulsive, and 46.2% combined. While no standard ADHD subtype distribution exists for adults, ratios in children have been reported as 3.6:1.3:2.2 for inattentive, hyperactive-impulsive, and combined types, respectively (64). Among individuals with ADHD and fibromyalgia, one-third are inattentive, and two-thirds are combined type (13). In this study, patients with BMS had a higher proportion of the hyperactive-impulsive type, potentially explaining their “impatient”, “active”, and “irritable” phenotypes, which may predispose them to seek invasive procedures or engage in doctor shopping.

**Figure 4:**
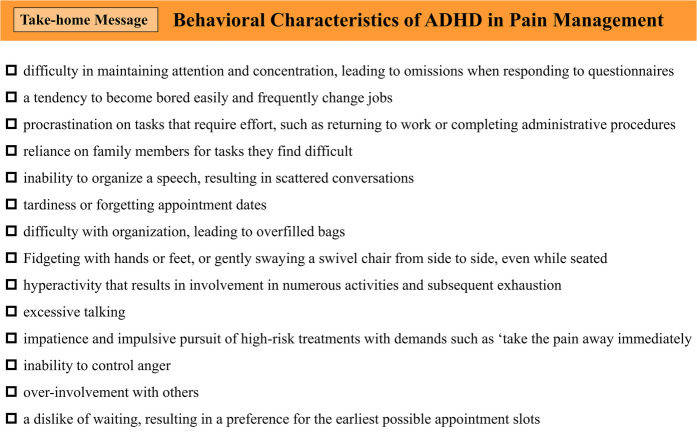
Behavioral characteristics of ADHD in pain management. ADHD, attention-deficit/hyperactivity disorder.

Based on our clinical experience and the list of ADHD behavioral characteristics outlined in DIVA 2.0 (40), we identified behavioral traits commonly observed in patients with ADHD during consultations for chronic pain conditions, such as BMS. Figure 4 shows a sample of ADHD behavioral characteristics that clinicians should be mindful of and assess during consultations. These characteristics underline the importance of ADHD screening in this population.

However, in dental and pain clinics that frequently treat patients with BMS, resources are often limited. In addition, the CAARS used in this study requires payment and consists of 66 questions, making it a challenging tool for routine screening. Therefore, when ADHD behavioral characteristics are observed in patients with BMS and comorbid ADHD is suspected, it is preferable to first use the Adult ADHD Self-Report Scale (ASRS) (65) for screening. ASRS is a free tool consisting of only six questions, making it a more feasible option for initial screening.

If a patient tests positive on ASRS, a consultation with a psychiatrist should be considered. However, ASRS has a sensitivity and specificity of 68.7% and 99.5% (65), respectively, indicating relatively low sensitivity and a possibility of false negatives. Therefore, even if ASRS results are negative, if a patient exhibits prominent ADHD behavioral traits, it may still be appropriate to consider the possibility of ADHD comorbidity and consult a psychiatrist while presenting the findings of this study.

The pharmacotherapy algorithm used in this study led to significant improvements in pain, anxiety, depression, and catastrophic thinking in treatment-resistant patients with BMS presenting with comorbid ADHD.

ADHD pathophysiology involves dopaminergic and noradrenergic dysfunctions (66). Medications in this study's algorithm enhanced dopamine and norepinephrine neurotransmission, likely improving both ADHD and pain symptoms. Dopamine and norepinephrine are key neurotransmitters in pain modulation (67), and their activation likely contributed to symptom improvement.

Traditionally, TCAs have been employed in the management of idiopathic orofacial pain, including BMS (68). Their mechanism of action involves norepinephrine reuptake inhibition, which enhances both norepinephrine and dopamine neurotransmission in the prefrontal cortex (69), potentially benefiting individuals with undiagnosed ADHD (21). Furthermore, ADHD medications such as MP and ATX have been shown to alleviate hyperalgesia and reduce pain thresholds through modulation of central sensitization (70–72). The high utilization of APZ and MP in this study underscores the role of enhanced dopaminergic neurotransmission in the nucleus accumbens (52), aligning with the dopamine dysfunction hypothesis of BMS pathophysiology (7).

Pre-treatment CBF-SPECT imaging revealed consistent patterns of regional hyperperfusion in the pgACC, insular cortex, precuneus, and PCC, alongside relative hypoperfusion in other frontal regions. Notably, reduced blood flow in the frontal lobe was observed in nine of 10 patients (90.0%). These findings are noteworthy as the pgACC and insular cortex are integral components of the pain matrix, while the precuneus and PCC are part of the DMN, which modulates internal cognitive processes related to external stimuli. Electrophysiological studies have shown that neuronal activity in the pgACC and PCC begins prior to voluntary reactions to noxious stimuli and persists beyond stimulus cessation, suggesting their involvement in perceiving pain stimuli as emotional experiences (73). Specifically, the pgACC has been implicated in responses to pathological skin stimuli (74) and ruminative thought processes (75), both of which can exacerbate the emotional experience of pain. We have previously demonstrated decreased precuneus CBF with chronic pain improvement following ADHD pharmacotherapy (35). ADHD medications in this study reduced activity in the DMN, likely attenuating pgACC hyperactivity and contributing to symptomatic improvement in BMS. Furthermore, reduced CBF in the frontal lobe has been identified as a potential biomarker distinguishing patients with ADHD from healthy individuals (76). Therefore, the SPECT findings in this study suggest that individuals with ADHD comorbid with BMS may exhibit physiological vulnerability to pain in the brain.

In this study, ADHD medications were associated with the normalization of CBF distribution, as shown in Figure 3 and Supplementary Table S1. Patients 4, 5, and 12 exhibited pronounced hyperperfusion in the pgACC and insula, while Patient 6, who lacked such hyperperfusion, maintained higher social and occupational functioning despite experiencing BMS.

This study has some limitations. First, the highly specific patient cohort drawn from a tertiary care center and the small sample size (*n* = 14) limit the generalizability of the findings. The participants in this study were unique in that they had proved difficult to treat even by dental specialists at a tertiary care university hospital; therefore, extrapolating these results to patients with BMS in more general settings, such as in typical dental clinics, should be performed with caution. The small sample size of only 14 participants means that the observed ADHD comorbidity rate of 92.9% may be a specific result limited to this study population. Furthermore, in this study, ADHD diagnoses were made by a single psychiatrist, posing a risk of overdiagnosis and diagnostic bias. Ideally, to ensure the validity of ADHD diagnoses, assessments by an additional psychiatrist should have been conducted. However, owing to limited human resources, this was not feasible. To compensate for this limitation, we utilized the CAARS-O, a paid assessment tool that provides an objective evaluation of ADHD symptoms as observed by significant others in daily life, rather than relying solely on free self-report ADHD scales (65). This approach allowed us to assess ADHD in a setting different from the clinical consultation environment. Second, the absence of a control group precludes definitive attribution of the observed improvements to the pharmacotherapy algorithm vs. spontaneous remission. Further studies involving larger, more diverse cohorts and randomized controlled designs while controlling for comorbid conditions other than ADHD are necessary to validate these findings. Third, among the participants in this study, 28.6% were classified as DYS and 35.7% as ID. Ideally, the implementation of family-involved operant behavioral therapy and assertiveness training as part of a multidisciplinary treatment approach could have further improved their symptoms (43). However, our pain center does not have a dedicated psychologist, making it impossible to implement these interventions. In the future, dentists will need to adopt a multidisciplinary approach, not only utilizing the ADHD treatment methods provided by psychiatrists, as demonstrated in this study, but also collaborating with clinical psychologists while considering patients' psychosocial factors. Furthermore, there have been reports on the effectiveness of homeopathic approaches, such as acupuncture and repetitive transcranial magnetic stimulation (37), which are alternative treatments for BMS that were not examined in this study. Therefore, these treatment options should also be explored in the future.

In conclusion, this study highlights the frequent comorbidity of ADHD in refractory BMS and its association with aberrant CBF patterns. It further demonstrates the potential of ADHD pharmacotherapy to ameliorate pain, cognitive dysfunction, and abnormal CBF in these patients. Given the high prevalence of undiagnosed ADHD in adults, routine screening for ADHD symptoms in patients with BMS using the CAARS-S/O is imperative. Collaborative, multidisciplinary approaches, incorporating other psychiatric expertise and targeted ADHD treatment, are essential to optimize outcomes in this complex population.

## Data Availability

The original contributions presented in the study are included in the article/Supplementary Material, further inquiries can be directed to the corresponding author.
