# Supplementary material for: Recognition and treatment of attention deficit-hyperactivity disorder in patients with treatment-resistant burning mouth syndrome: a retrospective case study
Source: Front Pain Res (Lausanne). 2025 Apr 23;6:1536584. doi: 10.3389/fpain.2025.1536584 (PMC12055801; doi:10.3389/fpain.2025.1536584)
Supplement: Supplementary file 1 [file Table1.docx]

Supplementary Material

# Supplementary Figures and Tables

## Supplementary Tables

**Table S1.** Patient characteristics and various clinical scores and findings

| No. | Sex | Age (years) | Pain duration (months) | Symptoms of attention-deficit | Symptoms of hyperactivity-impulsivity | ADHD subtype | Highest score in the sub-scales | | Psychiatric comorbidity | Drop out | Medication regimen (mg/day) | | | | | | Pain NRS maximum | | Pain NRS minimum | | Pain NRS average | | HADS-A | | HADS-D | | PCS | | CGI-S | SPECT | |
| --- | --- | --- | --- | --- | --- | --- | --- | --- | --- | --- | --- | --- | --- | --- | --- | --- | --- | --- | --- | --- | --- | --- | --- | --- | --- | --- | --- | --- | --- | --- | --- |
|  |  |  |  |  |  |  | CAARS-S | CAARS-O |  |  | MP | ATX | GF | APZ | VFX | DXT | Pre | Post | Pre | Post | Pre | Post | Pre | Post | Pre | Post | Pre | Post |  | Pre | Post |
| 1 | F | 40 | 164 | 8 | 7 | Combined type | 67** | 57 | ASD | － |  |  |  |  | 225 |  | 5 | 0 | 1 | 0 | 5 | 0 | 7 | 3 | 6 | 9 | 37 | 23 | 4 | Increased; pgACC, insular, precuneus.  Decreased; other frontal regions. | Frontal hypoperfusion improved slightly. |
| 2 | F | 76 | 48 | 1 | 6 | Hyperactive-impulsive type | 48 | 41 |  | + |  |  |  |  |  |  | 5 | N/A | 2 | N/A | 5 | N/A | 3 | N/A | 1 | N/A | 34 | N/A | 4 | Increased; pgACC, insular, precuneus.  Decreased; other frontal regions. | N/A |
| 3 | F | 75 | 264 | 8 | 5 | Combined type | 50 | 62* |  | + |  |  |  |  |  |  | 8 | N/A | 1 | N/A | 7 | N/A | 10 | N/A | 7 | N/A | 37 | N/A | 4 | Increased; precuneus. Decreased; frontal lobe | N/A |
| 4 | F | 38 | 24 | 4 | 6 | Hyperactive-impulsive type | 59 | 57 | ASD | － | 27 | 30 |  | 3 | 112.5 |  | 7 | 0 | 2 | 0 | 6 | 1 | 5 | 4 | 3 | 2 | 26 | 8 | 5 | Increased; pgACC, insular, precuneus.  Decreased; other frontal regions. | pgACC and insular slightly decreased. |
| 5 | F | 39 | 15 | 3 | 6 | Hyperactive-impulsive type | 65* | 57 | ASD | ＋ |  |  |  |  |  |  | 6 | N/A | 1 | N/A | 4 | N/A | 8 | N/A | 0 | N/A | 31 | N/A | 5 | Increased; pgACC, insular, precuneus.  Decreased; other frontal regions. | N/A |
| 6 | M | 44 | 84 | 0 | 5 | Hyperactive-impulsive type | 53 | 44 |  | － | 72 |  | 6 | 9 |  |  | 5 | 1 | 1 | 0 | 3 | 0 | 4 | 3 | 3 | 1 | 21 | 5 | 2 | Increased; pgACC, insular, precuneus. | pgACC and precuneus slightly decreased. |
| 7 | F | 42 | 6 | 9 | 6 | Combined type | 90** | 77** | Depression | － | 72 | 120 |  | 12 | 225 |  | 8 | 8 | 8 | 7 | 8 | 8 | 19 | 14 | 21 | 15 | 52 | 52 | 5 | N/A |  |
| 8 | F | 67 | 72 | 7 | 1 | Inattentive type | 64* | 52 |  | + |  |  |  |  |  |  | 3 | N/A | 3 | N/A | 3 | N/A | 8 | N/A | 7 | N/A | 21 | N/A | 4 | N/A |  |
| 9 | F | 59 | 60 | 5 | 3 | Inattentive type | 46 | 52 |  | － |  | 20 |  |  |  |  | 7 | 1 | 5 | 0 | 7 | 0 | 13 | 2 | 7 | 1 | 43 | 30 | 2 | N/A |  |
| 10 | F | 79 | 34 | 8 | 2 | Inattentive type | 85** | 81** |  | － | 36 |  |  | 6 |  |  | 10 | 0 | 10 | 0 | 10 | 0 | 15 | 8 | 20 | 15 | 49 | 39 | 4 | N/A |  |
| 11 | M | 74 | 96 | 9 | 8 | Combined type | 81** | 54 |  | － | 18 |  |  |  |  |  | 5 | 1 | 2 | 0 | 6 | 1 | 8 | 2 | 5 | 1 | 30 | 7 | 4 | Increased; pgACC, insular, precuneus.  Decreased; other frontal regions. | Frontal hypoperfusion improved slightly. |
| 12 | F | 78 | 120 | 1 | 2 | - | 50 | 54 | ASD | － |  |  |  |  |  | 20 | 3 | 2 | 2 | 1 | 4 | 2 | 7 | 6 | 6 | 7 | 15 | 16 | 5 | Increased; pgACC, insular, precuneus, PCC  Decreased; other frontal regions | N/A |
| 13 | F | 71 | 26 | 5 | 5 | Combined type | 50 | 50 |  | － |  |  |  | 2.5 |  |  | 5 | 1 | 4 | 0 | 5 | 0 | 8 | 8 | 7 | 10 | 17 | 15 | 2 | Increased; pgACC, insular, precuneus.  Decreased; other frontal regions. | Almost no change. |
| 14 | F | 58 | 10 | 6 | 6 | Combined type | 50 | 63* |  | － |  |  |  | 9 |  |  | 1 | 1 | 1 | 1 | 1 | 1 | 1 | 1 | 2 | 3 | 9 | 1 | 4 | Increased; pgACC, insular, precuneus.  Decreased; other frontal regions. | Frontal hypoperfusion improved slightly. |

*60<T-score≤65 (borderline level), **65<T-score (clinically significant level). ADHD, attention-deficit hyperactivity disorder; APZ, aripiprazole; ASD, autism spectrum disorder; ATX, atomoxetine; CAARS-S/O, Connors’ Adult ADHD Rating Scale Self-Report/Observer rated; CGI-S, clinical global impression severity; DXT, duloxetine hydrochloride; GF, guanfacine; HADS-A/D, Hospital Anxiety and Depression Scale - anxiety and depression ; MP, methylphenidate; N/A, not available; NRS, Numerical Rating Scale; pgACC, perigenual anterior cingulate cortex; PCC, posterior cingulate cortex; PCS, Pain Catastrophizing Scale; SPECT, single photon emission computed tomography ; VFX, venlafaxine hydrochloride
